# Role of PPAR-related genes in chronic heart failure: evidence from large populations

**DOI:** 10.1186/s12872-023-03554-8

**Published:** 2023-11-10

**Authors:** Zun-Ping Ke, Wen-Qi Tao, Gang Zhao, Kuan Cheng

**Affiliations:** 1 Department of Geriatrics, Shanghai Fifth People’s Hospital, Fudan University, Shanghai, China; 2 Department of Cardiology, Jing’an District Centre Hospital of Shanghai, Fudan University, Shanghai, 200040 China; 3 Department of Cardiology, Zhongshan Hospital, Shanghai Institute of Cardiovascular Diseases, Fudan University, Shanghai, China

**Keywords:** Heart Failure, PPAR, Microenvironment, Biological, Bioinformatics

## Abstract

**Background:**

The role of PPAR signaling and its associated genes in the pathogenesis and progression of chronic heart failure (CHF) remains elusive.

**Methods:**

We accessed the gene expression profile and relevant baseline information of CHF samples from the Gene Expression Omnibus (GEO) database, specifically from the GSE57338 project.

**Results:**

From GSE57338 project, we derived the expression value of 126 PPAR-related genes. A protein-protein interaction network was then established to illustrate potential protein interactions. ClueGO analysis results revealed that these genes predominantly participate in functions such as export across plasma membrane, regulation of lipid metabolic process, fatty acid metabolism, circulatory system vascular processes, alcohol metabolism, triglyceride metabolism and regulation of lipid localization and response to nutrient. Using the cytohubba plug-in in Cytoscape, we pinpointed ACADM, PPARG and CPT2 as potential central molecules in HF pathogenesis and progression. Subsequent Gene Ontology and Kyoto Encyclopedia of Genes and Genomes analysis delved into the potential biological role of these three genes in CHF. Immune infiltration analysis suggested that the infiltration level of neutrophils and M2 macrophages might be notably influenced by these genes, thereby playing a role in the CHF mechanism.

**Conclusions:**

Our research provides a comprehensive insight into the significance of PPAR associated genes in CHF development. Notably, the genes ACADM, PPARG and CPT2 emerged as potential targets for clinical interventions.

**Supplementary Information:**

The online version contains supplementary material available at 10.1186/s12872-023-03554-8.

## Introduction

Heart failure (HF) is characterized by the heart’s inability to efficiently pump the venous return blood volume due to compromised systolic and/or diastolic function. This leads to blood accumulation in the venous system and insufficient blood circulation in the arterial system. Current data suggests that the global incidence of HF ranges between 100 and 900 cases per 100,000 individuals annually [1]. HF can be categorized into acute HF and chronic HF (CHF), with CHF being the primary cause of cardiovascular-related mortalities. HF can be categorized into acute HF and chronic HF (CHF), with CHF being the primary cause of cardiovascular-related mortalities [2]. Hence, there remains a pressing need to identify effective treatments to enhance the survival prospects of CHF patients.

The peroxisome proliferator-activated receptors (PPARs) are nuclear hormone receptors that respond to fatty acids and their derivatives. As part of the ligand-activated subset of the nuclear hormone receptor family, PPARs encompass PPARα, PPARβ/δ, and PPARγ subtypes. These subtypes primarily regulate cellular metabolism and inflammation [3–5]. Numerous studies indicate a strong correlation between PPAR signaling and various diseases [6]. For instance, in hepatic steatosis, ETV5 modulates hepatic fatty acid metabolism through the PPAR signaling pathway, influencing disease progression [7]. The LncRNA TINCR/microRNA-107/CD36 regulatory axis operates within the context of colorectal cancer progression through the PPAR signaling pathway [8]. Additionally, in diabetic retinopathy, Fufang Xueshuantong alleviates disease progression by harnessing the PPAR signaling pathway to modify retinal hemodynamics and structure [9]. Significantly, PPAR signaling is pivotal in heart disease [4]. Evidence suggests that PGC1/PPARα signaling induces cardiomyocyte hypertrophy through YAP1 and facilitates cardiomyocyte contractility development via SF3B2 [10]. Activation of PPARγ signaling can counteract pulmonary hypertension and forestall right HF by promoting fatty acid oxidation [11]. A decline in PPARβ/δ expression in cardiac myocytes can suppress fatty acid transport and β-oxidation-related gene activity, leading to energy acquisition deficits in these cells [3]. Moreover, Wojtkowska et al. detected the PPAR expression in the aorta and left ventricle of 157 CAD patients who underwent CABG, finding that PPAR expression is not a reliable predictive factor for the development of HF in these patients post-surgery [12]. PPAR signaling is closely related to cardiac activity [13], and in this study, we will focus on the role of genes related to the PPAR signaling pathway in CHF.

The swift advancements in bioinformatics provide researchers with a powerful tool to delve deeper into understanding diseases [14]. In our study, we sourced the expression values of 126 PPAR-related genes from the GSE57338 expression matrix. Subsequently, we crafted a protein-protein interaction network to elucidate their potential protein interactions. Results from the clueGO analysis revealed a predominant enrichment of these genes in pathways such as export across the plasma membrane, lipid metabolic process regulation, fatty acid metabolism, circulatory system vascular processes, alcohol metabolism, triglyceride metabolism, lipid localization regulation, and response to nutrients. Employing the cytohubba plug-in within Cytoscape, we discerned that ACADM, PPARG, and CPT2 could be pivotal molecules in the genesis and progression of CHF. We further investigated their potential biological implications in CHF. Immune infiltration assessments suggested that the presence of neutrophils and M2 macrophages could be markedly influenced by these three genes, thereby playing a role in the CHF mechanism.

## Methods

### Acquisition of public data

Data on CHF was sourced from the open-access Gene Expression Omnibus (GEO) database under the GSE57338 project (313 individuals with/without CHF). This project provides RNA-seq data for both CHF and control samples. We accessed the gene expression profile directly through the “Series Matrix File(s)” link. Before commencing the analysis, we executed data preprocessing, which encompassed probe IDs annotation, filling in missing values, and data normalization.

### Acquisition of PPAR target genes

We derived the list of PPAR target genes from the PPAR gene website [15].

### Protein interaction network

The protein interaction network was established with the assistance of the STRING database [14]. For visualizing the network, we employed the Cytoscape software, and its cytohubba plug-in aided in pinpointing the hub nodes [16].

### Biological enrichment investigation

For biological enrichment analysis based on the input genes, we utilized the ClueGO app within the Cytoscape software [17]. The “clusterprofiler” package was employed to carry out both Gene Ontology (GO) and Kyoto Encyclopedia of Genes and Genomes (KEGG) analysis, adhering to the set threshold [18–21].

### Immune microenvironment

Immune microenvironment quantification in patients was executed using the CIBERSORT algorithm [22].

### Statistical analysis

All statistical analyses were conducted utilizing R software, with a significance threshold set at 0.05. Depending on data distribution characteristics, appropriate statistical methods were chosen.

## Results

### Role of PPAR target genes in CHF

**Fig. 1:**
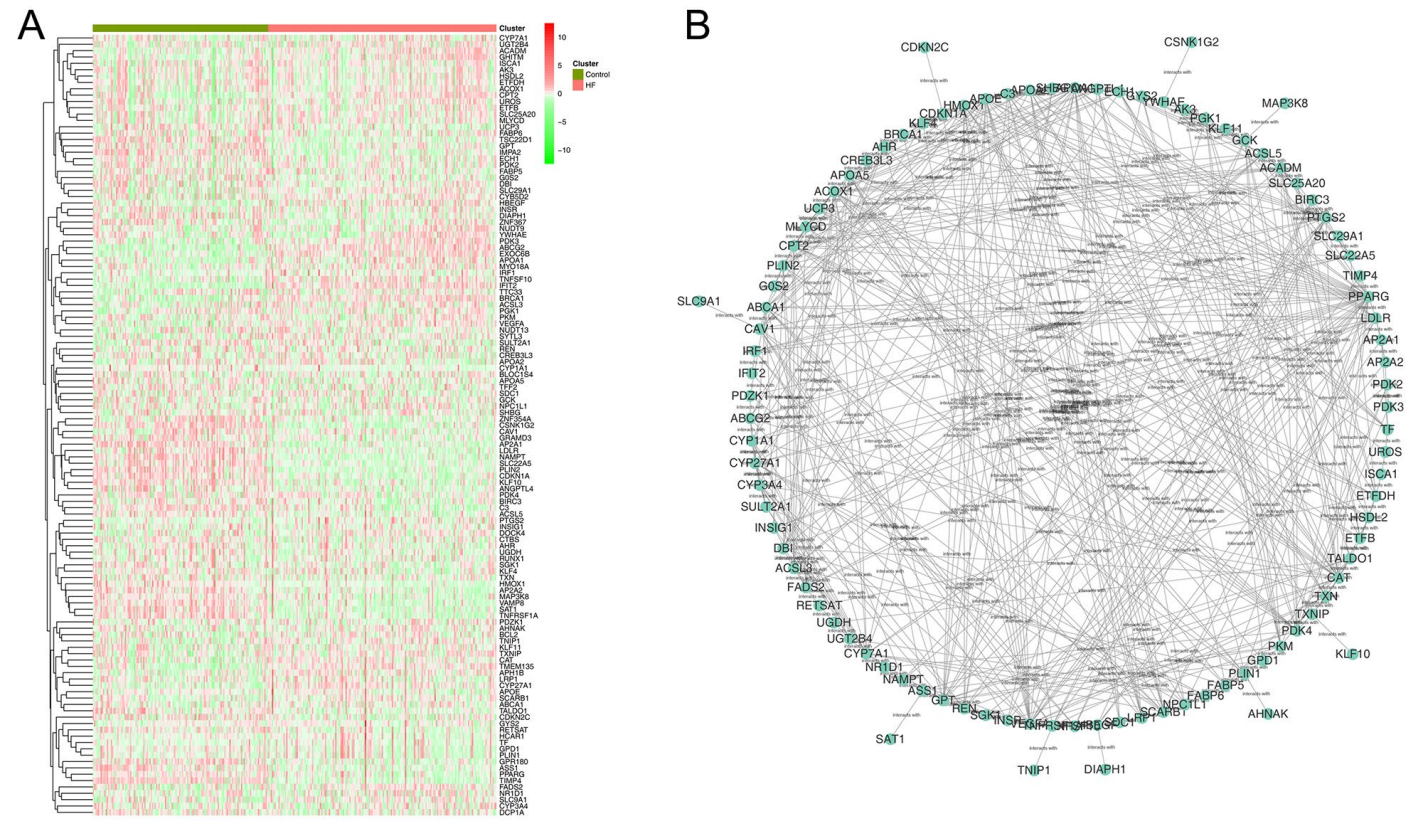
Role of PPAR target genes in CHF **Notes: A**: The expression pattern of PPAR target genes between CHF and control samples, which was shown in heatmap form; **B**: Protein interaction network of these PPAR target genes was constructed from the STRING database and visualized using the cytoscape software

**Fig. 2:**
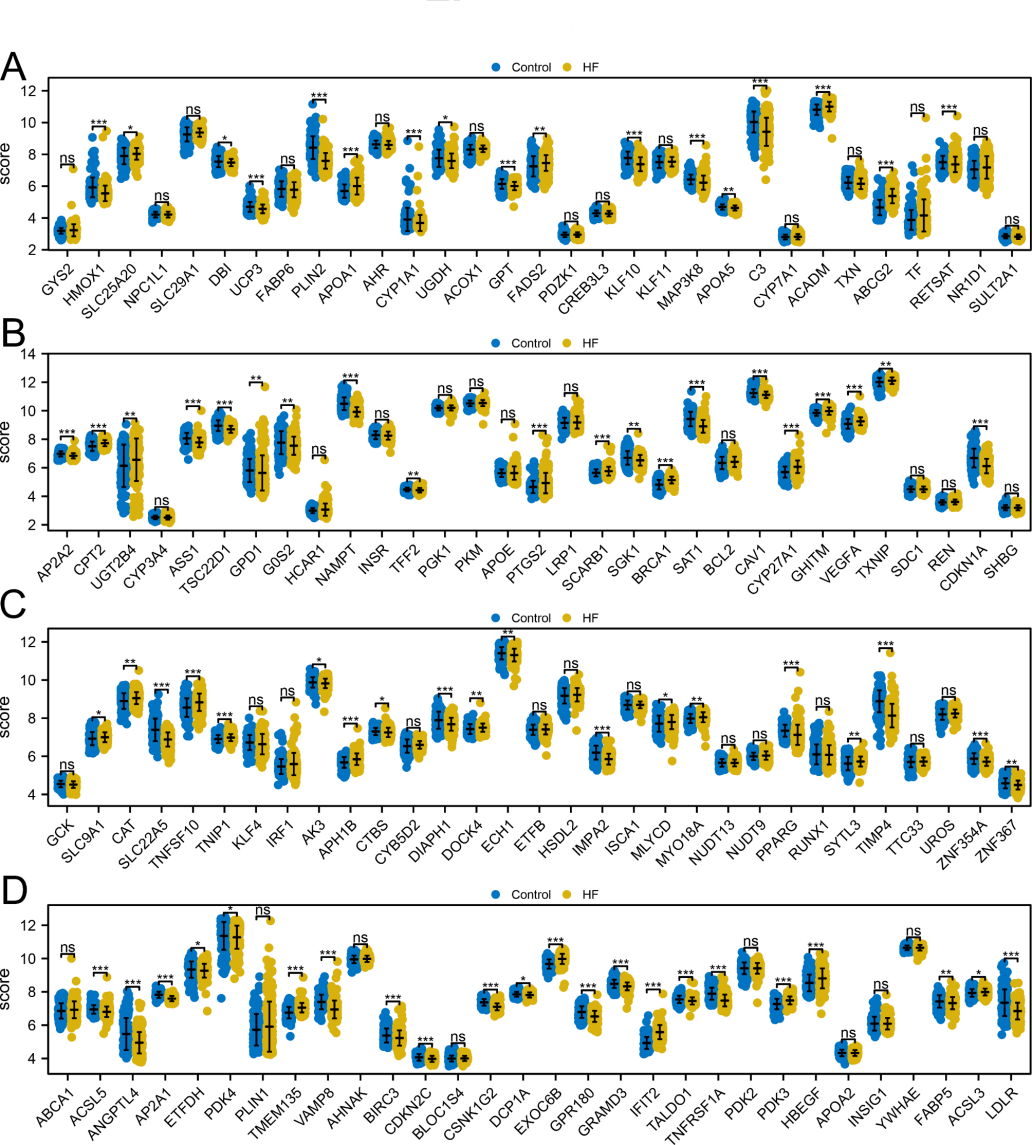
The expression level of PPAR target genes in CHF and control samples **Notes: A-D**: Expression level of PPAR target genes in CHF and control samples, ns = P < 0.05, * = P < 0.05, ** = P < 0.01, *** = P < 0.001

The flow chart of this study was shown in **Figure ****S1****.** Using the list derived from the PPAR gene database, we extracted expression values of PPAR target genes. Figure 1 A displays the expression trends of these genes in CHF and control samples. The protein interaction network for these PPAR target genes is depicted in Fig. 1B. Our results indicated that the PPAR target genes SLC25A20, APOA1, FADS2, ACADM, ABCG2, CPT2, UGT2B4, PTGS2, SCARB1, BRCA1, CYP27A1, GHITM, VEGFA, TXNIP, SLC9A1, CAT, TNFSF10, TNIP1, APH1B, DOCK4, MLYCD, MYO18A, SYTL3, TMEM135, EXOC6B, IFIT2, PDK3, HBEGF, ACSL3 were upregulated, while HMOX1, DBI, UCP3, PLIN2, CTP1A1, UGDH, GPT, KLF10, MAP3K8, APOA5, C3, RETSAT, AP2A2, ASS1, TSC22D1, GPD1, G0S2, NAMPT, TFF2, SGK1, SAT1, CAV1, CDKN1A, SLC22A5, AK3, CTBS, DIAPH1, ECH1, IMPA2, PPARG, TIMP4, ZNF354A, ZND367, ACSL5, ANGPTL4, AP2A1, ETFDH, PDK4, VAMP8, BIRC3, CDKN2C, CSNK1G2, DCP1A, GRP180, GRAMD3, TALDO1, TNFRSF1A, FABP5 were downregulated in the CHF tissue (Table 1; Fig. 2A-D).

**Table 1:** The role of PPAR target genes in HF

	HF vs. control samples		
Dataset	Upregulated	Downregulated	Not significant
GSE57338	SLC25A20, APOA1, FADS2, ACADM, ABCG2, CPT2, UGT2B4, PTGS2, SCARB1, BRCA1, CYP27A1, GHITM, VEGFA, TXNIP, SLC9A1, CAT, TNFSF10, TNIP1, APH1B, DOCK4, MLYCD, MYO18A, SYTL3, TMEM135, EXOC6B, IFIT2, PDK3, HBEGF, ACSL3	HMOX1, DBI, UCP3, PLIN2, CTP1A1, UGDH, GPT, KLF10, MAP3K8, APOA5, C3, RETSAT, AP2A2, ASS1, TSC22D1, GPD1, G0S2, NAMPT, TFF2, SGK1, SAT1, CAV1, CDKN1A, SLC22A5, AK3, CTBS, DIAPH1, ECH1, IMPA2, PPARG, TIMP4, ZNF354A, ZND367, ACSL5, ANGPTL4, AP2A1, ETFDH, PDK4, VAMP8, BIRC3, CDKN2C, CSNK1G2, DCP1A, GRP180, GRAMD3, TALDO1, TNFRSF1A, FABP5	GYS2, NPC1L1, SLC29A1, FABP6, AHR, ACOX1, PDZK1, CREB3L3, KIF11, CYP7A1, TXN, TF, NR1D1, SULT2A1, CYP3A4, HCAR1, INSR, PGK1, PKM, APOE, LRP1, BCL2, SDC1, REN, SHBG, GCK, KLF4, IRF1, CYB5D2, ETFB, HSDL2, ISCA1, NUDT13, NUDT9, RUNX1, TTC33, UROS, ABCA1, PLIN1, AHNAK, BLOC1S4, PDK2, APOA2, INSIIG1, YWHAE

### ClueGO analysis

**Fig. 3:**
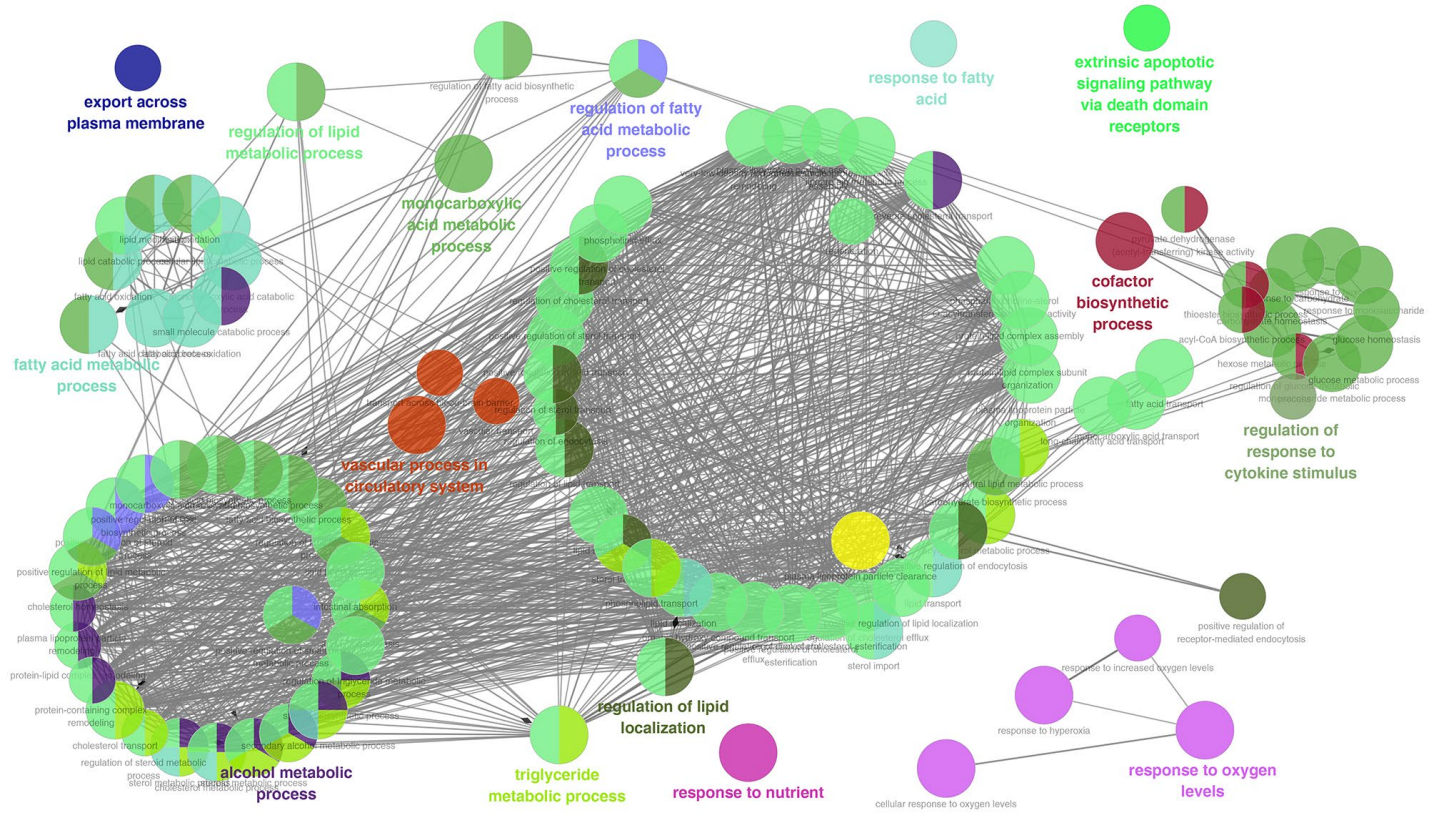
ClueGO analysis of the PPAR target genes **Notes**: ClueGO analysis was performed in the cytoscape software

Next, we delved into the potential biological impacts of these PPAR-related genes. ClueGO analysis results showed that these genes predominantly participated in pathways such as export across the plasma membrane, fatty acid metabolism, alcohol metabolism, lipid metabolic process regulation, vascular processes within the circulatory system, monocarboxylic acid metabolism, and triglyceride metabolism. Additionally, they were involved in regulating lipid localization, responding to nutrients, fatty acid metabolic process regulation, fatty acid response, cofactor biosynthesis, and adapting to oxygen levels (Fig. 3).

### Identification of the hub nodes and their biological role

**Fig. 4:**
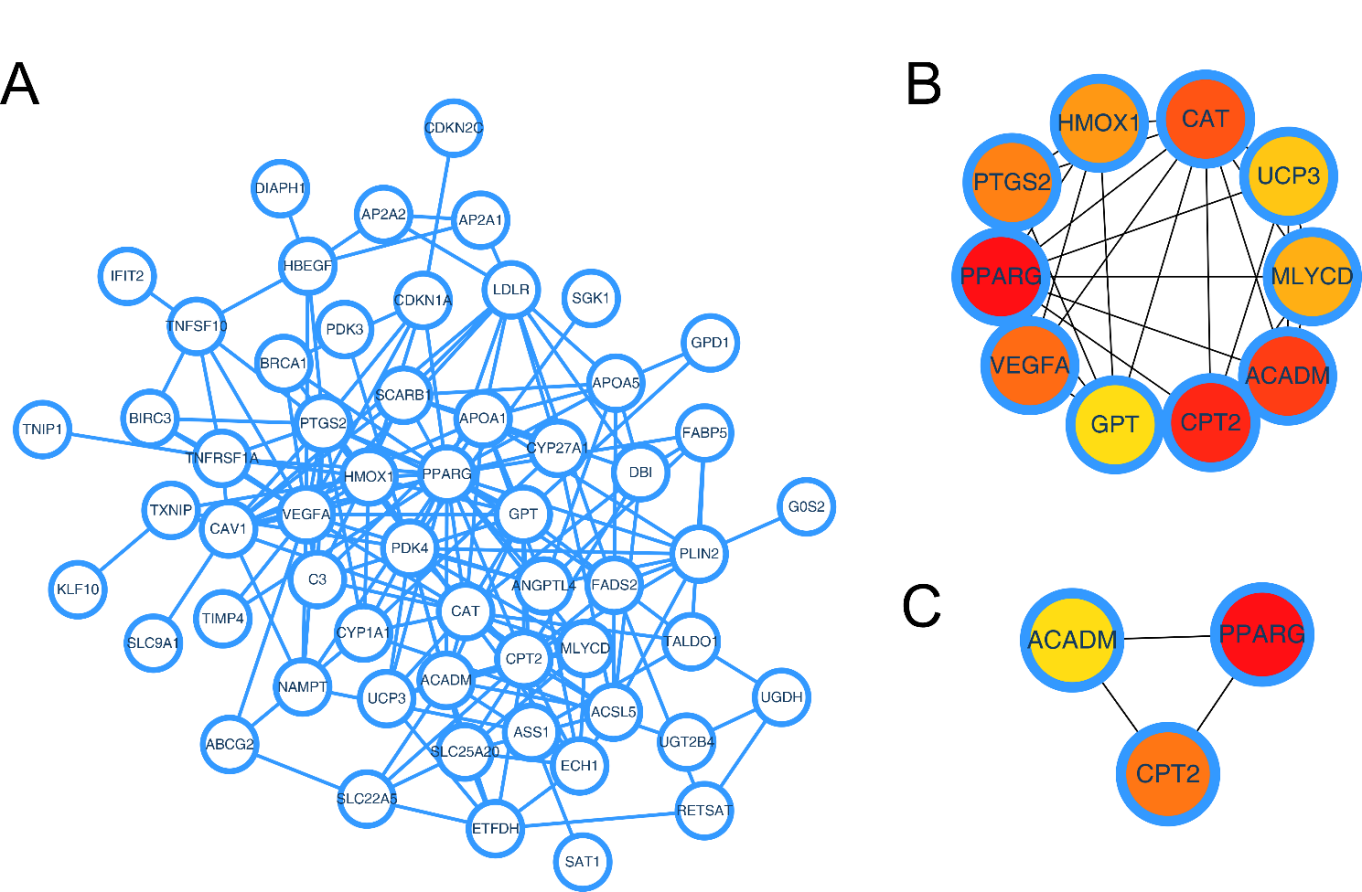
Protein interaction network of PPAR target genes differentially expressed between CHF and control samples **Notes: A**: Protein interaction network of these PPAR target genes differentially expressed between CHF and control samples; **B**: The top ten important nodes of the protein interaction network identified by cytoHubba plug-in of cytoscape software; **C**: The top three important nodes of the protein interaction network identified by cytoHubba plug-in of cytoscape software – ACADM, PPARG, CPT2.

Using the STRING database and Cytoscape software, we built a protein network centered on the PPAR target genes (Fig. 4A). The cytohubba software pinpointed the top 10 pivotal nodes as HMOX1, CAT, UCP3, MLYCD, ACADM, CPT2, GPT, VEGFA, PPARG, and PTGS2 (Fig. 4B), with ACADM, PPARG, and CPT2 emerging as the top three crucial nodes (Fig. 4C). Correlation analysis showed that there is a significant expression correlation between ACADM, PPARG, and CP2 pairwise (***Figure ******S2***, ACADM-PPARG: cor = -0.309, P < 0.001; ACADM-CPT2: cor = 0.427, P < 0.001; PPARG-CPT2: cor = -0.143, P = 0.011).

### The biological role of ACADM, PPARG and CPT2

**Fig. 5:**
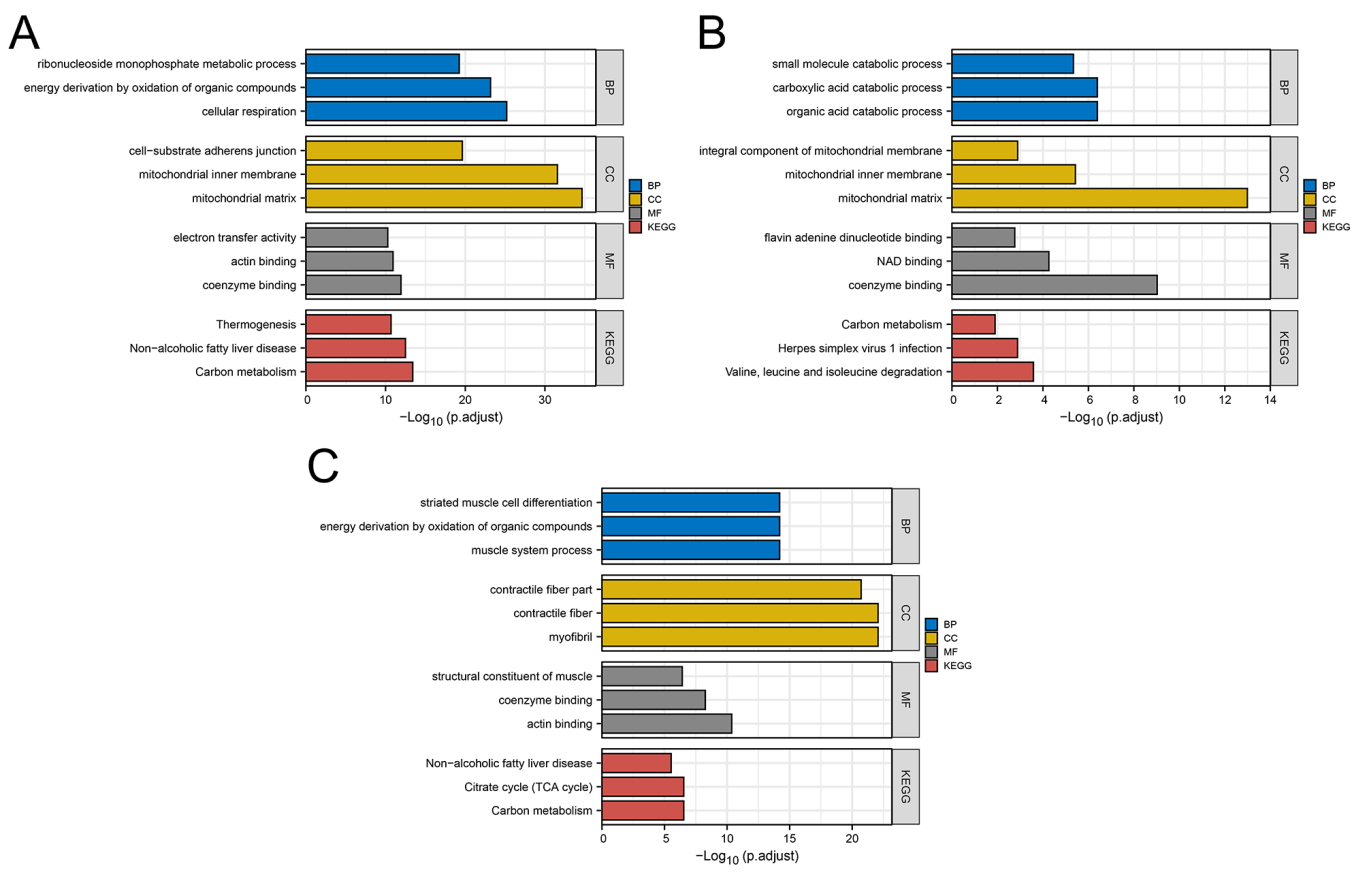
Biological enrichment analysis of ACADM, PPARG and CPT2 **Notes: A**: GO (BP, CC and MF) and KEGG analysis of ACADM; **B**: GO (BP, CC and MF) and KEGG analysis of CPT2; **C**: GO (BP, CC and MF) and KEGG analysis of PPARG.

Subsequently, we delved into the biological functions of ACADM, PPARG, and CPT2. Our findings revealed that ACADM played a significant role in ribonucleoside monophosphate metabolism, energy derivation from organic compound oxidation, cellular respiration, cell-substrate adherens junctions, mitochondrial inner membrane structuring, mitochondrial matrix processes, electron transfer activity, actin binding, coenzyme binding, thermogenesis, non-alcoholic fatty liver disease, and carbon metabolism (Fig. 5A); CPT2 was predominantly involved in small molecule catabolism, carboxylic and organic acid catabolic processes, integral components of the mitochondrial membrane, mitochondrial inner membrane dynamics, mitochondrial matrix, flavin adenine dinucleotide binding, NAD binding, coenzyme binding, carbon metabolism, herpes simplex virus 1 infection, and the degradation of valine, leucine, and isoleucine (Fig. 5B); PPARG was chiefly associated with striated muscle cell differentiation, energy derivation by organic compound oxidation, muscle system processes, contractile fiber components, myofibrils, muscle structural constituents, coenzyme and actin binding, non-alcoholic fatty liver disease, the citrate cycle (TCA cycle), and carbon metabolism (Fig. 5C).

### Effect of ACADM, PPARG and CPT2 CHF microenvironment

**Fig. 6:**
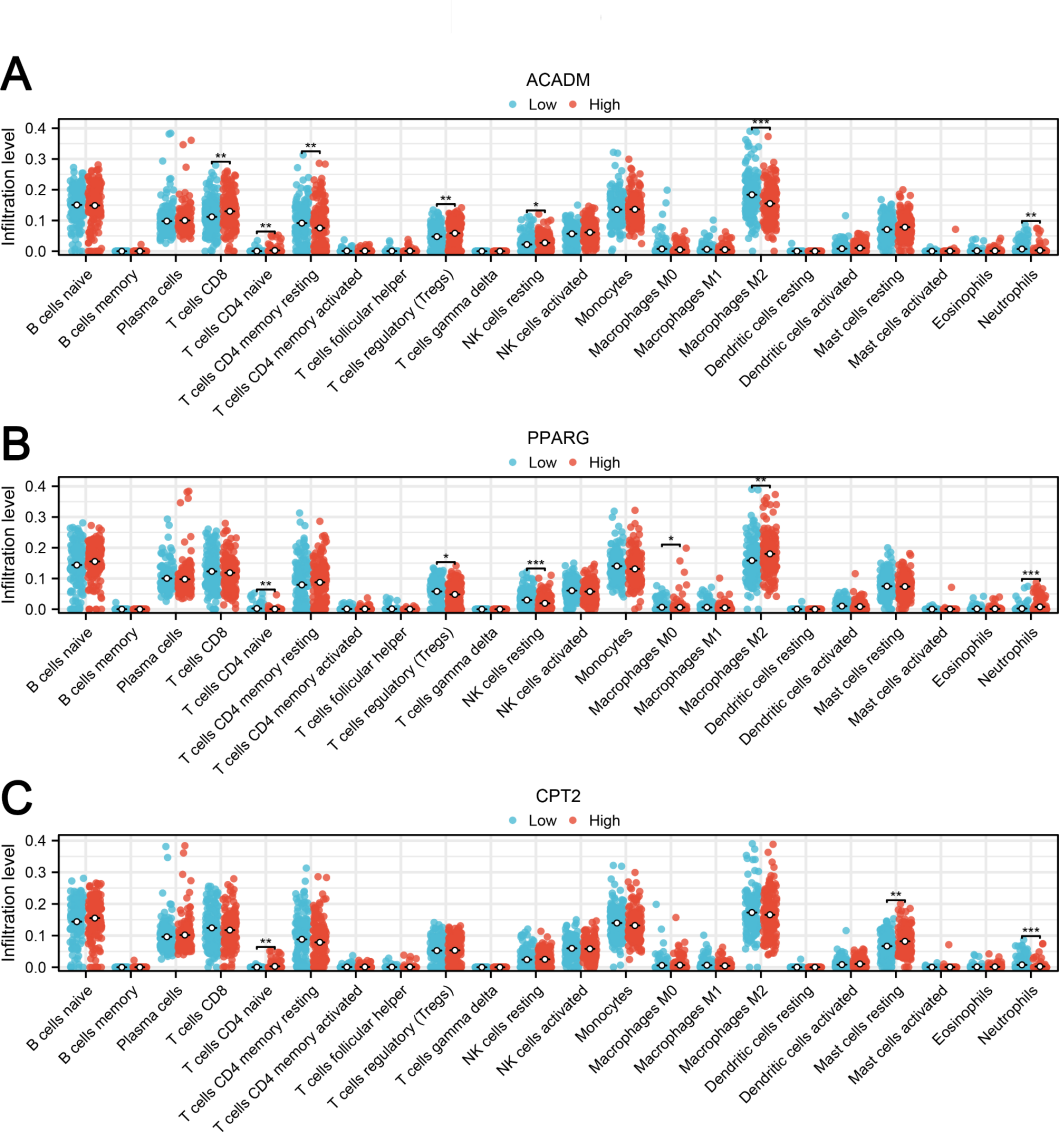
Immune microenvironment analysis using CIBERSORT algorithm **Notes: A**: Immune infiltration analysis of ACADM, * = P < 0.05, ** = P < 0.01, *** = P < 0.001; **B**: Immune infiltration analysis of PPARG, * = P < 0.05, ** = P < 0.01, *** = P < 0.001; **C**: Immune infiltration analysis of CPT2, ** = P < 0.01, *** = P < 0.001

We employed the CIBERSORT algorithm to assess the tissue microenvironment of CHF. The data suggested that patients exhibiting elevated ACADM expression displayed increased levels of CD8 + T cells, CD4 + naïve T cells, and Tregs, but decreased levels of resting CD4 + memory T cells, M2 macrophages, and neutrophils (Fig. 6A). Similarly, those with elevated PPARG expression manifested higher counts of naïve CD4 + T cells, Tregs, and resting NK cells, but a diminished count of M2 macrophages (Fig. 6B); Patients with augmented CPT2 expression showed increased levels of naïve CD4 + T cells and resting mast cells (Fig. 6C).

## Discussion

HF is a prevalent cardiovascular ailment characterized by cardiac insufficiency due to compromised systolic and/or diastolic heart function. Current data estimates that about 37.7 million individuals globally are affected by HF, with CHF standing out as the primary cardiovascular-related cause of mortality [1]. The significant threat CHF poses to human health amplifies the worldwide healthcare burden. Consequently, identifying effective treatments to enhance disease prognosis remains imperative.

In our research, we spotlighted HMOX1, CAT, UCP3, MLYCD, ACADM, CPT2, GPT, VEGFA, PPARG, and PTGS2 as potential central molecules in CHF’s evolution. These genes also wield significant influence over essential biological functions. HMOX1, for instance, offers protection against ischemic damage by stabilizing the hypoxia-inducible factor, HIF-1α [23]. Furthermore, HMOX1 exerts a protective effect against ischemia/reperfusion injury in cardiomyocytes [24]. Notably, studies show that HF can elevate cardiac UCP3 protein levels, which then instigates mitochondrial uncoupling, subsequently diminishing cardiac efficiency post high-frequency feeding [25]. CPT2, with its oxidative bioenergetic role, when deficient, can inhibit long-chain fatty acid oxidation, ushering in cardiac hypertrophy [26]. Within the dorsal aorta, VEGFA signaling can stimulate processes in endothelial, arterial, and hematopoietic stem cells [27]. Additionally, Qbestatine has been found to alleviate water retention in CHF by suppressing AQP2 expression in kidneys via PPARG signaling [28]. Research by Xing et al. has indicated that managing PTGS2 expression levels can mitigate symptoms of pulmonary hypertension [29]. Such findings underscore the significance of these identified genes in cardiovascular disorders, emphasizing their potential as therapeutic targets.

Immune infiltration analysis revealed that neutrophil and M2 macrophage infiltration might play a role in the progression of CHF. Research has demonstrated that elevated levels of neutrophils and persistent neutrophil activation are primary factors contributing to the excessive inflammation observed in acute HF and the long-term outcomes of CHF [30]. Hendrik B. Sager et al. identified macrophage proliferation in CHF following myocardial infarction [31]. Building on this, Michael Horckmans et al. elucidated that after a myocardial infarction, cardiomyocyte neutrophil depletion facilitates HF development and heart repair through the macrophage shift from the M1 to M2 phenotype [32]. Such insights highlight the potential of neutrophils and M2 macrophages as therapeutic targets in CHF management.

The clueGO analysis results indicated that the genes were primarily associated with processes such as export across the plasma membrane, regulation of lipid metabolism, fatty acid metabolism, vascular activities in the circulatory system, alcohol metabolism, triglyceride metabolism, lipid localization regulation, and nutrient response. It’s well-documented that a failing heart exhibits metabolic anomalies, predominantly favoring fatty acid metabolism [33]. Crucially, essential fatty acid metabolism plays a pivotal role in hemodynamic stress-induced myocardial phospholipid remodeling and is closely linked to cardiac contractile dysfunction [34]. Furthermore, the downregulation of genes involved in fatty acid metabolism has been tied to post-infarction HF in rats [35]. In terminal HF stages, a decline in myocardial lipid content paired with heightened ketone body utilization from lipid metabolism by cardiac cells has been observed [36]. Vascular biological processes are also intricately tied to HF. Research has shown that while myocardial angiogenesis can trigger myocardial hypertrophy, a hypertrophic response in the myocardium can also induce angiogenesis [37]. It’s well-established that alcohol consumption has a significant correlation with cardiovascular diseases [38]. The myocardium lacks alcohol dehydrogenase, making it susceptible to the direct toxic effects of ethanol and acetaldehyde on cardiac myocytes. Moreover, alcohol can suppress the activities of tricarboxylic acid cycle enzymes in mitochondria, impacting the energy uptake in cardiomyocytes, which can ultimately diminish their contractility [39].

Our study does have certain limitations. Firstly, aside from the GSE57338 cohort, the sample size in other CHF cohorts is quite limited, making it challenging to corroborate our findings in additional cohorts. Secondly, real-world validation of our results remains essential.

### Electronic supplementary material

Below is the link to the electronic supplementary material.

Supplementary Material 1

Supplementary Material 2

Supplementary Material 3

## Data Availability

The original data of GSE57338 can be obtained from https://www.ncbi.nlm.nih.gov/geo/query/acc.cgi?acc=GSE57338. All data are available from the corresponding author on reasonable request.

## Abbreviations

chronic heart failure: CHF
Gene Expression Omnibus: GEO
Heart failure: HF
peroxisome proliferator-activated receptors: PPARs
Gene Ontology: GO
Kyoto Encyclopedia of Genes and Genomes: KEGG
