# Supplementary figures and images for: Role of PPAR-related genes in chronic heart failure: evidence from large populations
Source: BMC Cardiovasc Disord. 2023 Nov 10;23:552. doi: 10.1186/s12872-023-03554-8 (PMC10638691; doi:10.1186/s12872-023-03554-8)

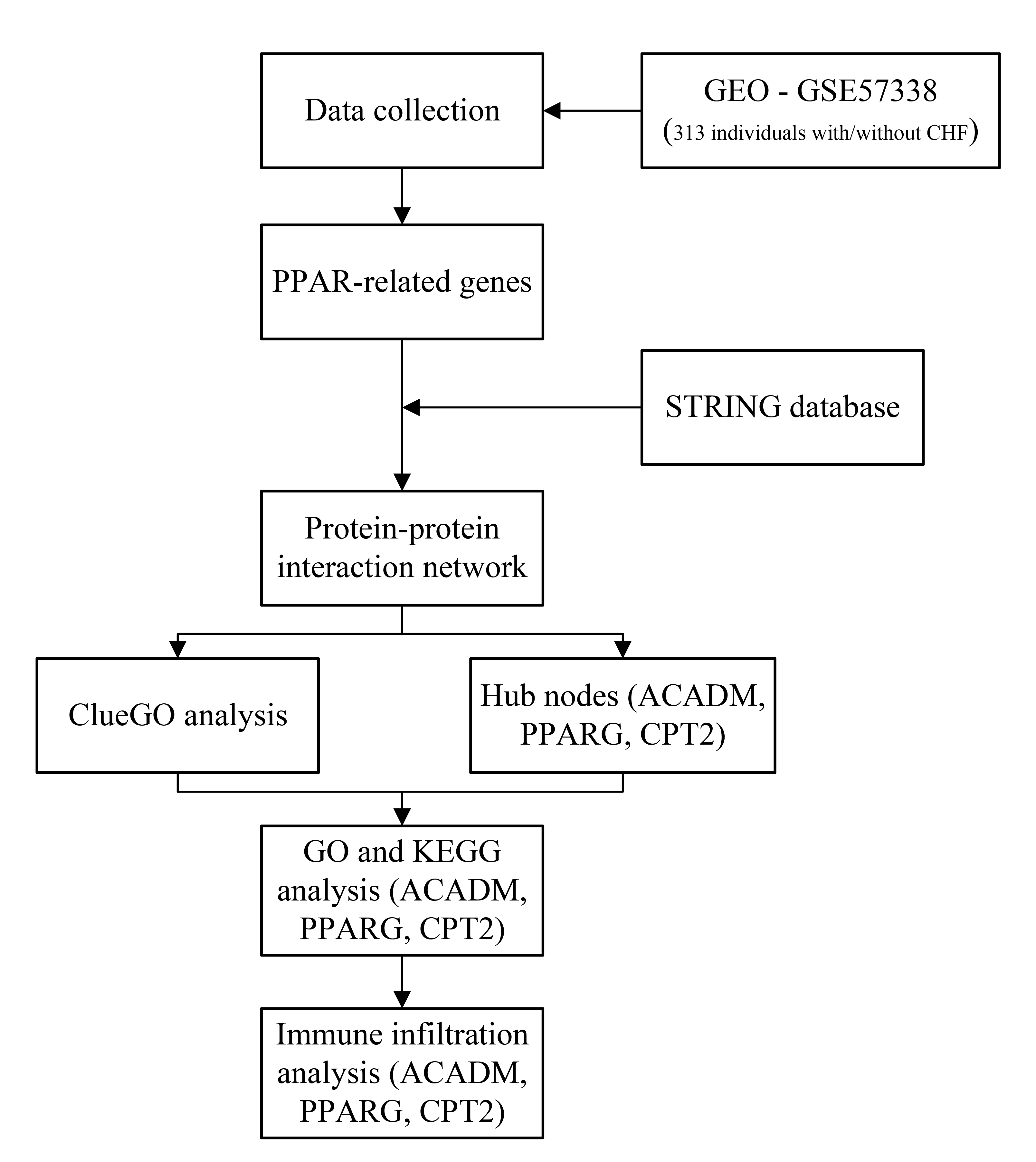

Supplement: Supplementary file 1 — Supplementary Material 1 [file 12872_2023_3554_MOESM1_ESM.tif]

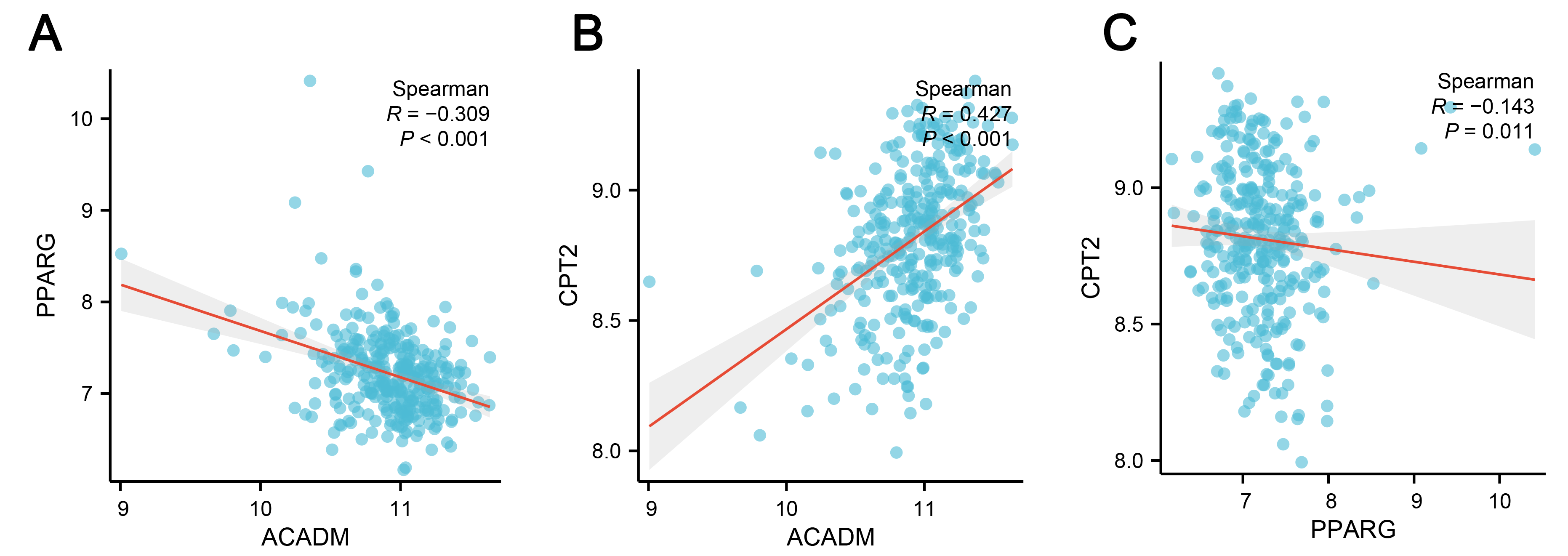

Supplement: Supplementary file 2 — Supplementary Material 2 [file 12872_2023_3554_MOESM2_ESM.tif]
