# Supplementary material for: Role of PPAR-related genes in chronic heart failure: evidence from large populations
Source: BMC Cardiovasc Disord. 2023 Nov 10;23:552. doi: 10.1186/s12872-023-03554-8 (PMC10638691; doi:10.1186/s12872-023-03554-8)
Supplement: Supplementary file 3 — Supplementary Material 3 [file 12872_2023_3554_MOESM3_ESM.docx]

**Figure legends**

**Figure S1. The flow chart of our study**

**Figure S2. The mRNA correlation between ACADM, PPARG and CPT2**
